# Supplementary material for: Five-Year Cardiovascular Outcomes after Infective Endocarditis in Patients with versus without Drug Use History
Source: J Pers Med. 2022 Sep 22;12(10):1562. doi: 10.3390/jpm12101562 (PMC9605539; doi:10.3390/jpm12101562)
Supplement: Supplementary file 1 [file jpm-12-01562-s001.zip › jpm-1835822-supplementary.pdf]

Supplemental Table S1. Cumulative 5-year outcomes before propensity score matching

Outcome	PWUD (N = 15,573) Cumulative Incidence	Non-PWUD (N = 49,521) Cumulative Incidence	HR (95% CI)	Log-rank *P*
All-cause mortality				
Cumulative 5-year	25.3%	18.0%	1.34 (1.28-1.41)	< 0.001
<1 Year	11.9%	9.1%	1.21 (1.13-1.28)	< 0.001
1-3 Years	9.1%	5.4%	1.63 (1.48-1.80)	< 0.001
3-5 Years	7.4%	4.3%	1.73 (1.47-2.03)	< 0.001
VT/VF	6.7%	6.2%	1.04 (0.94-1.16)	0.437
Myocardial infarction	9.8%	6.1%	1.59 (1.46-1.78)	< 0.001
Heart failure	19.6%	16.5%	1.17 (1.09-1.24)	< 0.001
Atrial fibrillation/flutter	9.4%	11.7%	0.70 (0.64-0.76)	< 0.001
Ischemic stroke	8.5%	5.5%	1.41 (1.29-1.55)	< 0.001
Intracranial hemorrhage	4.6%	2.2%	1.87 (1.64-2.13)	< 0.001

Incidence calculated with Kaplan-Meier estimator. Abbreviations. CI: confidence interval; HR: hazard ratio PWUD: people who use drugs; VT/VF: ventricular tachycardia/ventricular fibrillation.

Supplemental Table S2. Cumulative 5-year outcomes after propensity score matching in patients treated surgically

Outcome	PWUD (N = 392) Cumulative Incidence	Non-PWUD (N = 392) Cumulative Incidence	HR (95% CI)	Log-rank *P*
All-cause mortality	33.8%	24.8%	1.26 (0.91-1.75)	0.165
VT/VF	15.0%	8.0%	1.37 (0.75-2.52)	0.305
Myocardial infarction	15.1%	7.0%	1.85 (0.97-3.52)	0.059
Heart failure	29.8%	34.4%	0.92 (0.58-1.46)	0.720
Atrial fibrillation/flutter	19.2%	22.9%	0.82 (0.53-1.28)	0.376
Ischemic stroke	13.7%	11.7%	1.07 (0.59-1.95)	0.818
Intracranial hemorrhage	6.3%	6.6%	1.04 (0.49-2.21)	0.922

Incidence calculated with Kaplan-Meier estimator. Abbreviations. CI: confidence interval; HR: hazard ratio PWUD: people who use drugs; VT/VF: ventricular tachycardia/ventricular fibrillation.

Supplemental Table S3. Cumulative 5-year outcomes after propensity score matching in patients treated with medical therapy only

Outcome	PWUD (N = 6,390) Cumulative Incidence	Non-PWUD (N = 6,390) Cumulative Incidence	HR (95%CI)	Log-rank *P*
All-cause mortality	25.6%	22.5%	1.06 (0.97-1.16)	0.179
VT/VF	6.1%	5.3%	1.14 (0.93-1.39)	0.221
Myocardial infarction	9.2%	6.2%	1.38 (1.15-1.66)	0.001
Heart failure	16.6%	14.8%	1.13 (0.99-1.30)	0.080
Atrial fibrillation/flutter	8.9%	9.2%	0.94 (0.79-1.12)	0.493
Ischemic stroke	7.4%	6.4%	1.15 (0.97-1.39)	0.136
Intracranial hemorrhage	4.0%	2.4%	1.39 (1.07-1.82)	0.015

Incidence calculated with Kaplan-Meier estimator. Abbreviations. CI: confidence interval; HR: hazard ratio PWUD: people who use drugs; VT/VF: ventricular tachycardia/ventricular fibrillation.

Supplemental Table S4. Cumulative 5-year outcomes after propensity score matching in male PWUD vs. non-PWUD

Outcome	PWUD (N = 4,472) Cumulative Incidence	Non-PWUD (N = 4,472) Cumulative Incidence	HR (95% CI)	Log-rank *P*
All-cause mortality	27.8%	26.0%	0.99 (0.90-1.10)	0.865
VT/VF	8.5%	7.4%	1.15 (0.93-1.44)	0.196
Myocardial infarction	9.8%	8.2%	1.17 (0.95-1.44)	0.140
Heart failure	20.3%	17.7%	1.24 (1.06-1.44)	0.007
Atrial fibrillation/flutter	12.8%	11.9%	0.93 (0.78-1.11)	0.422
Ischemic stroke	7.9%	7.7%	1.11 (0.90-1.37)	0.348
Intracranial hemorrhage	5.2%	3.6%	1.34 (1.01-1.78)	0.044

Incidence calculated with Kaplan-Meier estimator. Abbreviations. CI: confidence interval; HR: hazard ratio PWUD: people who use drugs; VT/VF: ventricular tachycardia/ventricular fibrillation.

Supplemental Table S5. Cumulative 5-year outcomes after propensity score matching in female PWUD vs. non-PWUD

Outcome	PWUD (N = 3,717) Cumulative Incidence	Non-PWUD (N = 3,717) Cumulative Incidence	HR (95% CI)	Log-rank *P*
All-cause mortality	24.8%	20.9%	1.15 (1.02-1.30)	0.019
VT/VF	5.7%	5.5%	1.07 (0.82-1.41)	0.624
Myocardial infarction	9.1%	6.0%	1.45 (1.14-1.84)	0.002
Heart failure	19.2%	15.1%	1.24 (1.05-1.47)	0.010
Atrial fibrillation/flutter	7.6%	8.6%	0.89 (0.71-1.12)	0.318
Ischemic stroke	8.3%	5.2%	1.60 (1.25-2.04)	< 0.001
Intracranial hemorrhage	3.7%	2.6%	1.21 (0.87-1.69)	0.259

Incidence calculated with Kaplan-Meier estimator. Abbreviations. CI: confidence interval; HR: hazard ratio PWUD: people who use drugs; VT/VF: ventricular tachycardia/ventricular fibrillation. In comparison of the hazard ratios, women had significantly higher stroke risk (P = 0.026 for the difference of HRs), and borderline higher mortality risk (P=0.062 for the difference of HRs).

Supplemental Table S6. Cumulative 5-year outcomes after propensity score matching in White/Caucasian PWUD vs. non-PWUD

Outcome	PWUD (N = 4,769) Cumulative Incidence	Non-PWUD (N = 4,769) Cumulative Incidence	HR (95% CI)	Log-rank *P*
All-cause mortality	23.6%	21.5%	1.05 (0.94-1.17)	0.408
VT/VF	6.4%	4.9%	1.30 (1.03-1.65)	0.030
Myocardial infarction	8.3%	5.7%	1.42 (1.14-1.77)	0.002
Heart failure	19.4%	12.9%	1.46 (1.25-1.70)	< 0.001
Atrial fibrillation/flutter	9.7%	9.2%	0.99 (0.82-1.20)	0.927
Ischemic stroke	7.5%	5.8%	1.29 (1.04-1.60)	0.022
Intracranial hemorrhage	4.2%	2.6%	1.49 (1.10-2.01)	0.009

Incidence calculated with Kaplan-Meier estimator. Abbreviations. CI: confidence interval; HR: hazard ratio PWUD: people who use drugs; VT/VF: ventricular tachycardia/ventricular fibrillation.

Supplemental Table S7. Cumulative 5-year incidence after propensity score matching in Black/African American PWUD vs. non-PWUD

Outcome	PWUD (N = 1,717) Cumulative Incidence	Non-PWUD (N = 1,717) Cumulative Incidence	HR (95% CI)	Log-rank *P*
All-cause mortality	31.2%	32.2%	0.88 (0.76-1.02)	0.089
VT/VF	9.9%	8.0%	1.08 (0.78-1.49)	0.650
Myocardial infarction	14.0%	11.0%	1.28 (0.97-1.70)	0.083
Heart failure	25.2%	26.8%	0.96 (0.76-1.21)	0.714
Atrial fibrillation/flutter	11.8%	14.3%	0.76 (0.57-0.99)	0.046
Ischemic stroke	12.6%	8.5%	1.67 (1.22-2.28)	0.001
Intracranial hemorrhage	4.9%	4.4%	0.99 (0.65-1.53)	0.982

Incidence calculated with Kaplan-Meier estimator. Abbreviations. CI: confidence interval; HR: hazard ratio PWUD: people who use drugs; VT/VF: ventricular tachycardia/ventricular fibrillation. In comparison of the hazard ratios, White/Caucasian PWUD had higher heart failure risk (P=0.003 for the difference of HRs), and borderline higher mortality risk (P=0.059 for the difference of HRs).
